# Hallmarks of Cancers: Primary Antibody Deficiency *Versus* Other Inborn Errors of Immunity

**DOI:** 10.3389/fimmu.2021.720025

**Published:** 2021-08-17

**Authors:** Hassan Abolhassani, Yating Wang, Lennart Hammarström, Qiang Pan-Hammarström

**Affiliations:** ^1^Division of Clinical Immunology, Department of Biosciences and Nutrition, Karolinska Institutet, Huddinge, Sweden; ^2^Division of Clinical Immunology, Department of Laboratory Medicine, Karolinska University Hospital Huddinge, Karolinska Institutet, Stockholm, Sweden; ^3^Research Center for Immunodeficiencies, Pediatrics Center of Excellence, Children’s Medical Center, Tehran University of Medical Science, Tehran, Iran

**Keywords:** inborn errors of immunity, primary immunodeficiency, predominantly antibody deficiency, hallmarks of cancer, immune dysregulation, genome instability, chronic inflammation

## Abstract

Inborn Errors of Immunity (IEI) comprise more than 450 inherited diseases, from which selected patients manifest a frequent and early incidence of malignancies, mainly lymphoma and leukemia. Primary antibody deficiency (PAD) is the most common form of IEI with the highest proportion of malignant cases. In this review, we aimed to compare the oncologic hallmarks and the molecular defects underlying PAD with other IEI entities to dissect the impact of avoiding immune destruction, genome instability, and mutation, enabling replicative immortality, tumor-promoting inflammation, resisting cell death, sustaining proliferative signaling, evading growth suppressors, deregulating cellular energetics, inducing angiogenesis, and activating invasion and metastasis in these groups of patients. Moreover, some of the most promising approaches that could be clinically tested in both PAD and IEI patients were discussed.

## Introduction

Inborn Errors of Immunity (IEI), formerly known as primary immunodeficiencies, comprise at least 450 inherited diseases, from which selected patients manifest a frequent and early incidence of malignancies (1–3). As the main presentation, IEI patients are prone to recurrent infections (due to bacterial, viral, and parasitic agents) that predispose individuals to a chronic increase in inflammatory mediators, contributing to neoplasia (e.g., reactive oxygen and nitrogen intermediates, prostaglandins, and inflammatory cytokines). The longer the inflammation persists due to inadequate or inappropriate treatments, the higher the risk of associated tumorigenesis and the survival advantage of a cancerous cell (4). However, several other intrinsic and extrinsic causes of malignancies have been identified in both IEI-associated hematologic and solid tumors (5, 6). Considering the heterogeneous pathogenesis of IEI, different mechanisms underlying tumorigenesis in these patients would be expected. From an oncologic point of view, the main hallmarks of cancer have recently been suggested to dissect the complexity of neoplastic disease (7). The presented review compares oncologic hallmarks and the molecular defects underlying primary antibody deficiencies (PADs) with other IEI-associated cancers. The current published literature collection highlights that PAD patients have more diverse hallmarks of cancers compared to other IEIs (except combined immunodeficiency and immune dysregulation) and have a higher number of cases with heterogeneous genetic defects or unknown molecular etiologies. Of note, several therapeutic options are currently available for these diverse pathogeneses in PAD patients with cancer susceptibility, which should be considered by clinical immunologists and treating physicians.

## Avoiding Immune Destruction

The ability of recognition and elimination of developing tumors in the absence of external therapy, which is known as cancer immunosurveillance, can be defective in certain types of IEIs (8, 9). Although the overall increased relative risk of cancer in IEI patients is less than twofold, a skewed spectrum of cancers (mainly lymphoid malignancies in males) can result from different gene defects (10). Innate and adaptive cytotoxicity against pre-malignant or malignant cells can be affected by mutations associated with dysfunction of natural killer (NK) and CD8^+^ T cells (1). Therefore, the intrinsic genetic defects affecting the development or function of T cell (presenting with combined immunodeficiency, major histocompatibility complex class I deficiency, or hyper IgE syndromes) and NK cell (GATA2, MCM4, and FCGR3A deficiencies) may lead to cancer, in particular carcinomas (11–15). Familial hemophagocytic lymphohistiocytosis patients with mutations in *UNC13D, PRF1, STXBP2*, and *STX11* also present a significant defect in cytotoxicity, causing lymphoproliferative diseases and oncogenesis (16, 17).

Moreover, a proportion of patients with diseases of immune dysregulation show an increased susceptibility to herpes virus infections (mainly Epstein–Barr virus [EBV]-induced lymphoproliferative complications and malignancies), which resulted from defects in co-stimulatory molecules essential for CD8^+^ memory T-cell formation (e.g., CD27 and CD70 deficiencies and OX40 deficiency associated with higher risk of lymphoma and sarcoma) (18–21). Several other genes coordinate CD8^+^ T-cell activation and memory generation *via* various mechanisms, and therefore, mutations in these genes can increase the risk for developing EBV-associated lymphomas: controlling T-cell receptor-stimulated Mg2^+^ influx and concentrations (magnesium transporter 1, *MAGT1* gene) (22), modulating the SH2 domain-mediated interactions in signaling lymphocyte activation molecule (SLAM)-mediated activation (SH2 domain-containing 1A, *SH2D1A* gene) (23), sustaining the proliferation of activated lymphocytes by *de novo* mutations in genes associated with the pyrimidine synthesis pathway (nucleotide cytidine 5′ triphosphate synthases1, *CTPS1* gene) (24), activation of MAP-kinase cascade *via* guanine-nucleotide-exchange factors (RAS guanyl-releasing protein 1, *RASGRP1* gene) (25), and mediating critical signals from the T-cell receptor and activated lymphocyte-specific protein tyrosine kinase (interleukin-2-inducible T-cell kinase, *ITK* gene) (26). Although the mechanism of cancer immunosurveillance has been suggested in a minority of PAD with functional T cell defects, some EBV-associated cancer due to monogenic IEI can affect B cell terminal development and also present with antibody deficiency and lack of specific immunoglobulin production mimicking common variable immunodeficiency (CVID)-like phenotype (27, 28).

## Genome Instability and Mutation

Monogenic diseases of chromosome instability and DNA repair defects affecting steps of detection, removal, or further modification of the damaged DNA, and resynthesis and ligation of DNA strands can predispose both to immunodeficiency and cancer (29). T- B- receptor rearrangements [V(D)J recombinations] require the non-homologous end joining (NHEJ) pathway to process/repair double-strand DNA breaks and loss of function in various components of the NHEJ machinery present with T- B- severe combined immunodeficiency (SCID) (30, 31). In patients treated with hematopoietic stem cell transplantation, or carrying hypomorphic mutations in NHEJ factor encoding genes, survival may be associated with the development of hematological cancers, carcinomas, and sarcomas (5). Patients with DNA repair defects have a higher risk of EBV infections since the encoded viral proteins are implicated in the deregulation of DNA damage response signaling pathways (32). EBV infection disturbs ATM-mediated response (during the G2/M cell cycle *via* LMP1 and EBNA3C nuclear antigens) consistent with more frequent detection of EBV early antigen antibodies in patients with ataxia-telangiectasia in whom the incidence of lymphoma is increased (33, 34). Moreover, EBV attenuates DNA-dependent protein kinase and Artemis activities by depleting the p350/DNA-PK catalytic subunit and interacting with EBNA2, leading to a markedly increased incidence of EBV-induced lymphoproliferation in patients with pathogenic mutations in the *PRKDC* and *DCLRE1C* genes, up to 50% (34, 35).

Class switch recombination (CSR) and somatic hypermutation in peripheral B cells have a role in increasing the diversity of immunoglobulin classes as well as affinity maturation, which is accomplished by a large number of proteins involved in NHEJ, base excision repair, and mismatch repair (36). Ataxia-telangiectasia, Nijmegen breakage syndrome, Bloom’s syndrome, and constitutional mismatch repair deficiency (CMMRD) syndromes are the main immunodeficiencies within this category and the patients usually develop lymphomas (5, 37). Activation-induced cytidine deaminase (AICDA) and uracil DNA glycosylase (UNG) deficiencies specifically affect the CSR in B cells, presenting as a PAD known as hyper IgM syndrome with an increased incidence of hematologic cancers (38, 39).

Dysregulations in epigenetic modifications and chromatin remodeling may result in genomic instability and syndromic features mainly in the immunological and neurological systems (40). Genes underlying immunodeficiency with centromeric instability and facial anomalies (ICF) syndrome are responsible for DNA methylation and critical epigenetic modification during lymphocyte development, chromatin structure remodeling, and physiological DNA breaks (41). ICF patients display DNA hypomethylation mainly affecting satellite 2 and 3 repeats of pericentromers, which is very common in cancer cells (42), in line with the higher incidence of cancers in these patients (43). Of note, cases with ICF syndrome due to hypomorphic mutations may manifest without facial and neurologic symptoms, mimicking CVID-like phenotype with only antibody defects or recurrent infections and they may survive longer with a higher chance for the development of cancers (44, 45).

## Enabling Replicative Immortality

This cancer hallmark is described as an independently driven process involving the elongation of telomeres by reactivation of telomerase reverse transcriptase and increasing the cell proliferative capacity (46, 47). This process is regulated by the catalytic subunit of telomerase reverse transcriptase (TERT) that connects this hallmark to metabolic reprogramming, apoptosis, and tumor invasion (48). Thus, TERT and its associated elements could directly connect the various hallmarks of cancer (49). Dyskeratosis congenita (DKC) is a complex of syndromic features caused by defects in these proteins, which can result in a severe form of Hoyeraal–Hreidarsson syndrome due to short telomeres and genome instability (50–52). Recently, Coats-plus syndrome with mutations in *STN1* and *CTC1* have been described and linked to immunodeficiency with abnormal telomeres. This group of genetic abrogations frequently predisposes patients to myelodysplasia and leukemia (53, 54). Intriguingly, several cases of dyskeratosis congenita can show specifically with the initial presentation of antibody deficiency, and due to misclassification, they are more prone to the development of long-term complications like malignancies (27, 55).

## Tumor-Promoting Inflammation

Although chronic inflammation occurs in most IEI patients with a delayed diagnosis and poor treatment, some subgroups of patients can develop unrestrained systemic inflammatory reactions despite immunomodulation, which may lead to cancer (56, 57). This cancer hallmark is well characterized in disturbance of immune regulation with colitis (due to a defective IL10-STAT1 pathway) (58, 59) and predisposition to mucocutaneous candidiasis (mainly due to a defective IL17 pathway) (60) that can increase the susceptibility to lymphoma and carcinoma, respectively.

Moreover, CVID, as the most common symptomatic form of antibody deficiency, also had a higher rate of chronic inflammation despite regular and appropriate treatment (61). Due to its high prevalence, the majority of IEI cancer patients have a clinical diagnosis of CVID (10). CVID is a heterogeneous disease, and there is an ongoing debate about criteria for diagnosis that mainly rely on the fulfillment of specific immunologic criteria. Therefore, CVID is considered as an umbrella term constituting several different humoral immune failures and antibody production impairment due to unknown monogenic, polygenic, or epigenetic defects (27, 28). However, the main suggested pathogeneses for the cancer phenomenon in CVID patients are immune dysregulation and chronic infection due to lack of mucosal immunity (absence of IgA in selected CVID patients). Therefore, subsequent inflammation might be a tumor-predisposing factor especially towards gastric cancers in CVID cases (62–65). The same phenomenon can be present in other entities of PAD with low IgA levels including congenital agammaglobulinemia and IgA deficiency (62, 66–68). Of note, a minority of CVID patients can present chromosomal radiosensitivity due to disruption of DNA repair machinery and must be considered for tumorigenesis due to genome instability and regular screening for cancer and avoidance of malignancy triggers must be added to their routine management (69, 70).

## Resisting Cell Death

Autoimmune lymphoproliferative syndrome (ALPS) is the porotype of IEI, which is associated with apoptosis defects and malignancies (71). The most well-established activity of affected proteins in the FAS–FAS ligand and Caspase pathway is to mediate the apoptotic death of either virus-infected cells or cancer cells when engaged by a cytotoxic lymphocyte (72). Although lymphoma has been reported as the most common type of malignancy seen in these patients, additional types of cancers in this population suggest a broader cancer predisposition as previously observed with somatic *FAS* mutations (73–76). Since apoptosis has an important role in the development, function and maintenance of the immune system, it controls the duration of immune responses to foreign antigens and deletion of auto-reactive T and B lymphocytes (77). Similarly, several abnormalities in T- and B-cell apoptosis in patients with humoral immunodeficiencies such as CVID have been reported and suggested to be underlying the higher rate of malignancy, particularly lymphoma, in this group of patients (78, 79).

## Sustaining Proliferative Signaling

Self-sufficiency in growth signals, bypassing various checkpoints, may be implicated in a vast number of patients with IEI and cancers (80). Immune system defects and dynamical compensation in physiological circuits lead to increased production of stimulatory factors mainly in patients with stem cell and myeloid developmental defects (81). Congenital neutropenias and other syndromic IEI (Wiskott-Aldrich, Shwachman-Diamond, MonoMac and immuno-osseous dysplasia syndromes) affecting early non-lymphoid stem cell lineages can manifest with myelodysplasia and leukemia (82).

Higher rates of diagnosis during recent years and detailed follow-up of autosomal dominant gain-of-function defects in signal transducer and activator of transcriptions (STAT) (83, 84), caspase recruitment domain family members (CARD) (85, 86) and NACHT, LRR, and PYD domain-containing proteins (NLRP) have shown an increased incidence of both hematological and solid tumors (87). Of note in the PAD category, several gain-of-function genetic defects in the signaling of phosphoinositide 3-kinase (PI3K) and nuclear factor κ-enhancer of activated B cells (NF-κB) have been shown to be involved in the dysregulation of the adaptive immune response and continuous lymphoid tissue growth, thus increasing the susceptibility to lymphoma (88–92). Of note, a minority of patients with NF-κB defects also presented avoiding cellular immune destruction mainly due to abrogated CD8 T-cell immunity (93, 94).

## Evading Growth Suppressors

The diverse functions of tumor suppressors vary from proliferation restriction to the regulation of regenerative processes in different human cell types (95). However, these elements modulate the proliferation and differentiation of immune cells to protect their genomic integrity during physiologic cellular metabolic and proliferative stress (96). The existence of multiple tumor suppressor family members (e.g., p53, retinoblastoma, and Hippo genes) may allow certain family members to have taken on specific roles in the enhancement of hematopoietic stem cell regeneration, DNA repair, chromosome remodeling, and cell-cycle checkpoint for selecting the desired modification (97).

One of the main tumor suppressor pathways conferring immunodeficiency and susceptibility to cancers is the posttranslational regulation of phosphatase and tensin homolog (PTEN) (98). PTEN is a negative regulator of PI3K signaling and is very commonly mutated in human cancers. Since PTEN is essential during early development, only heterozygous loss-of-function mutants have been reported in individuals with CVID-like phenotype with lymphoproliferation and hyperplasia (99). The prototypical tumor suppressor gene and pathway is p53, which is also a key pathway component affected in a majority of DNA repair defects associated with immunodeficiency and cancers (e.g., patients with *ATM* and *MRE11* mutations) (100).

Dedicator of cytokinesis 8 (DOCK8) can act as a tumor suppressor in non-hematopoietic tissues by directly affecting apoptosis through regulation of migration, morphology, adhesion, and growth of cells, apart from its probable role in CD8^+^ T cells for tumor surveillance (101). Cytotoxic T lymphocyte-associated antigen 4 (CTLA4) is upregulated in activated naïve T cells through the T-cell receptor and subsequent engagement of the costimulatory receptor CD28 (102). This suppressive molecule acts as co-inhibitory and mutation in the autosomal dominant form impairs the function of regulatory T cells, thus increasing the risk for autoimmunity, chronic inflammation, and cancers (103). Patients with lipopolysaccharide-responsive and beige-like anchor protein (LRBA) deficiency, a crucial molecule for recycling of CTLA4 and the function of regulatory T cells, present a similar CVID-like phenotype with the development of both hematological and solid tumors (104, 105).

## Deregulating Cellular Energetics

IEIs associated with sustaining proliferative signaling induce endoplasmic reticulum stress, unfolded protein response, destabilization of mitochondrial membrane potentials, and disturbed energy metabolism (106, 107). Recent findings also suggest that there may be a common pathogenic mechanism that connects a high prevalence of cancer, metabolic disorders, atherosclerotic cardiovascular disease, and insulin-resistant diabetes in carriers of some DNA repair defects, in particular *ATM* mutations (108). Mutations of genes related to NHEJ and IEI disorders associated with chronic inflammation result in age-associated pathological conditions due to their roles in metabolic regulation in response to DNA damage avoiding further genomic instability (109, 110).

These defects in DNA repair and uncontrolled inflammation may induce stem cell exhaustion, cellular senescence, immunosenescence, low-grade chronic inflammation, activation of PI3K signaling, defective autophagy, and mitochondrial genome instability. It has been shown that ATM-dependent stress and dysregulation of inflammatory pathways mediate predisposition to both the metabolic syndrome and cancer (111).

## Inducing Angiogenesis

A series of well-orchestrated cellular adaptations occur to stimulate angiogenesis and enhance the survival of tumors in hypoxic conditions (112). Gain-of-function somatic mutations in RAS-associated genes (*KRAS* and *NRAS*) can result in RAS-associated autoimmune leukoproliferative disease (RALD) with lymphocytosis and lymphoproliferation, a phenocopy of ALPS (113, 114). The affected proteins are GTPases that serve as a signaling switch molecule, coupling receptor activation by specific growth factors with downstream effector pathways. After cancer-related hypoxia responses, in patients with RALD, the production of vascular endothelial growth factor (VEGF) is enhanced (115). Therefore, the over-activation of RAS signaling significantly stimulates angiogenesis and blocks apoptosis in hypoxic conditions (116).

Furthermore, in cancers associated with defective innate or adaptive immune responses, the balance between pro- and anti-angiogenic factors is perturbed by dysregulated cytokine production by innate immune cells (117). Increased inflammatory mediators as a consequence of antibody deficiency, diseases of immune dysregulation, and autoinflammatory diseases contribute to neoplasia by stimulation of angiogenesis, where a change confers a survival advantage to a tumor cell (56, 118). Therefore, the promotion of angiogenesis in the IEI tumors accelerates the migration of endothelial cells and formation of new blood vessels, and distorted and enlarged vascular architecture with increased permeability and irregular blood flow (119).

## Activating Invasion and Metastasis

A selected group of IEIs faces aggressive oncogenic risks due to an increased susceptibility for viral replication and persistence (120). Among those, transforming viral infections with a distant invasion have been reported by human papillomavirus (HPV infection in Epidermodysplasia verruciformis and WHIM syndrome) (121, 122) and herpes viruses family (particularly EBV susceptibility in immune dysregulation diseases). Of note, both groups of patients with HPV (mainly WHIM syndrome) and EBV infection susceptibility can mimic the phenotype of CVID-like due to their predominance of humoral immunodeficiency. Although both HPV and EBV oncoviruses have undertaken different powerful anti-apoptotic and proliferative programs, they can directly induce metastasis in infected tumor cells. In HPV-associated IEIs, E6 and E7 proteins can contribute to tumor invasion by impacting epithelial-to-mesenchymal transition (123, 124), while in EBV infection, the LMP2A protein can promote differentiation, survival, and cell growth by activating the PI3K pathway and pathways mediating cell mobility and invasion (125).

## Future Directions and Concluding Remarks

**Figure 1:**
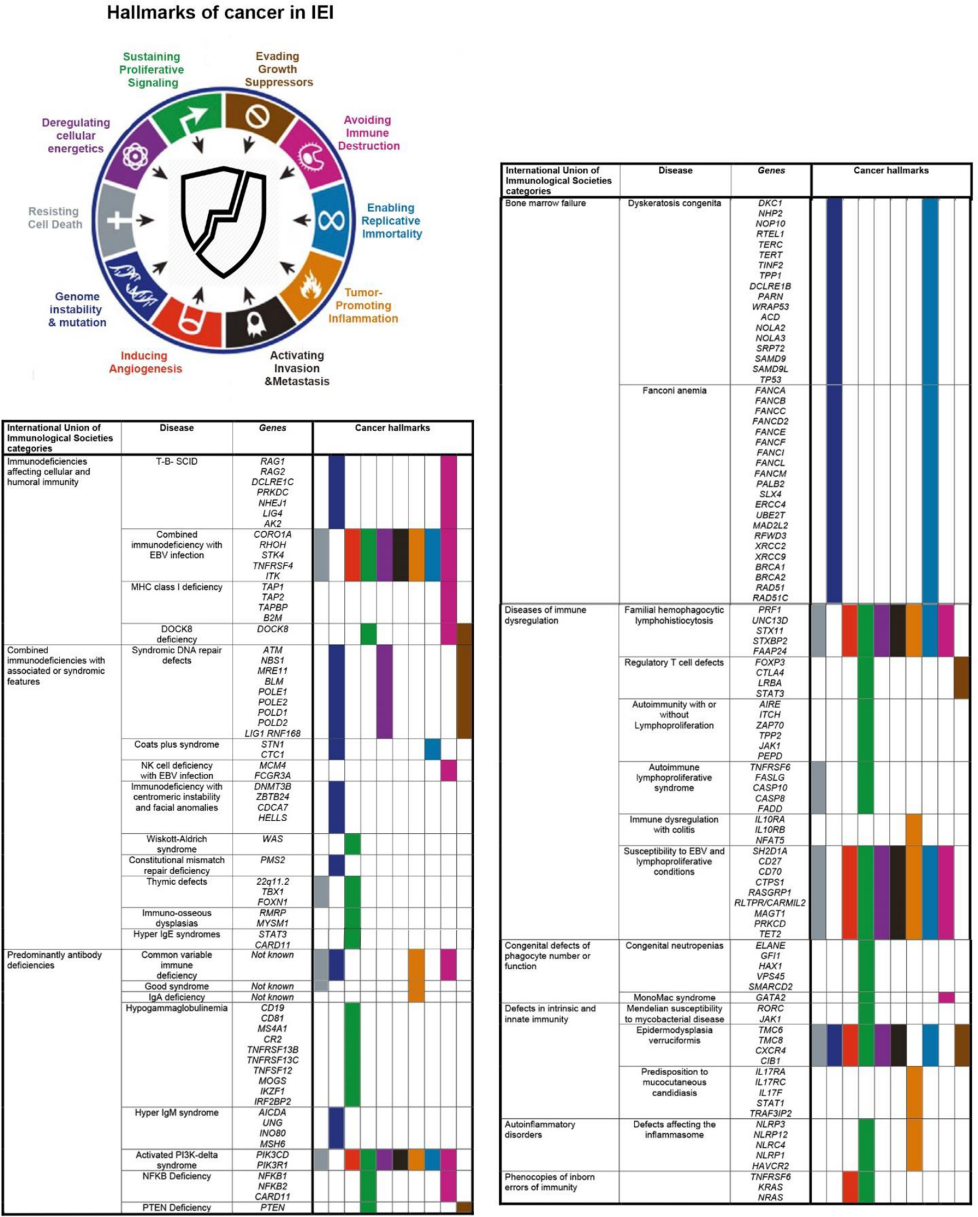
Cancer hallmark activation in different types of monogenic inborn errors of immunity (IEI) according to the International Union of Immunological Societies classification (1, 3).

The evaluation of the hallmarks of cancer in IEI patients helps to explain the multistep nature of oncogenesis in different forms of immune defects/dysfunction (**Figure 1**). This outlines the complexity of the development of cancer in each entity of IEIs, requiring the progressive acquisition of different necessary cellular hallmarks that constitute a malignant phenotype. The distribution of distinct types of cancers in patients with specific genetic defects highlights the cell-specific predisposition to an intrinsic cause or extrinsic exposure in the context of the genetic background of the host and the selective pressures imposed by the tissue microenvironment. The analysis of a cancer hallmark model would also facilitate understanding about the process of IEI carcinogenesis to relevant treatment. Recently, cancer hallmarks have been reorganized into seven updated compact parameters (126). It has been suggested to consider altered stress response favoring overall survival by combining defects of genome instability and mutation, enabling replicative immortality, tumor-promoting inflammation, and resisting cell death hallmarks (126). Moreover, a new hallmark for abetting microenvironment has been offered to cover cancer etiologies related to communication between the dynamic microenvironment of the affected organ and stromal cells (5, 126). IEI genes underlying each hallmark might help to investigate whether these newly proposed revisions are functionally and molecularly relevant.

Based on several lines of evidence, PAD patients constitute the highest proportion of IEI cases affected by malignancies. Moreover, several monogenic defects with different involved cancer hallmarks can mimic the clinical and immunologic phenotypes of PAD patients, mainly CVID. The abovementioned overview about IEI-induced and PAD-induced cancers indicated that these malignancies are amenable to immune prophylaxis by vaccines, prophylactic radiation limitation, and, most recently, targeted therapy. However, future clinical efforts in preventing or treating gene-specific-associated malignancies represent a combination of antiviral therapies, agents that induce cytotoxicity events, agents that improve DNA repair machinery, and agents that are used to successfully treat cancers with antagonists and agonists for IEI tumor stimulators and repressors. **Table 1** illustrates some of the most promising approaches that could be clinically tested in both PAD and IEI patients. Of note, other monogenic IEIs mainly with combined immunodeficiency and immune dysregulation also have diverse cancer hallmarks as PAD patients; however, they are more likely to be transplanted due to the risk of cancer, whereas most PADs may not be transplanted. The treatment of cancers in the context of immune defects, however, remains challenging and a detailed molecular investigation and multi-omics analysis of both germline and somatic (tumor) genome may increase the number of potential therapeutic targets and also further provide clues of potential resistance to therapy.

**Table 1:** Therapeutic and preventive approaches successfully used or potentially can be implemented to prevent primary immunodeficiency-associated cancer hallmarks.

Hallmark or Process	Agent or Vause	Drug or Modality
**Avoiding immune destruction**	EBV infection**	EBV-specific cytotoxic T lymphocytes
	Costimulatory agonist	Anti-GITR, anti-ICOS, anti-OX40, and anti-CD27
	Regulatory T cells**	Anti-CD25
**Deregulating cellular energetics**	Immunometabolism	IDO1 inhibitors, A2AR antagonists, Arginase inhibitors, and Glutaminase inhibitors
**Evading growth suppressors**	Dual checkpoint blockade*	Anti-CTLA-4 (Ipilimumab), anti-PD1 (Nivolumab), anti-PDL1 (Atezolizumab), anti-TIM3, anti-LAG3, anti-TIGIT, and anti-VISTA
**Genome instability and mutation**	DNA repair defect*	Decrease radiation exposure
	Epigenetic changes*	DNMT inhibitors and HDAC inhibitors
**Inducing angiogenesis**	RAS-associated autoimmune leuko-proliferative disease	Cetuximab, Pantitumumab, and Bevacizumab
**Sustaining proliferative signaling**	EBV infection**	Butyrate and Ganciclovir
	HPV infection*	L1 virus-like particles vaccine
	BTK activation*	Ibrutinib and Acalabrutinib
	PI3K activation**	Rifampicin, Buparlisib, Alpelisib, Nemiralisib, and Idelalisib
	PI3K or NFKB activation**	Rituximab, Ibritumomab Tiuxetan, and Tositumomab
	mTOR activation**	Everolimus
	MAPK/ERK activation**	Trametinib
	Stem cell and myeloid development defects	Bone marrow transplantation, CSF1R inhibitor, and HDAC inhibitors class IIa
	Cytokines	JAK inhibitors, TGF inhibitors, and MET inhibitors
**Tumor-promoting inflammation**	*H. pylori* infection*	Standard triple therapy consisting of proton pump inhibitor, clarithromycin, and amoxicillin
	Chronic inflammation*	Nonsteroidal anti-inflammatory drugs

EBV, Epstein–Barr virus; GITR, glucocorticoid-induced TNFR-related protein; ICOS, Inducible T-cell COStimulator; IDO1, Indoleamine 2;3-dioxygenase 1; A2AR, Adenosine 2A receptor; CTLA4, Cytotoxic T-lymphocyte protein 4 precursors; TIM3, T-cell immunoglobulin and mucin domain 3; LAG3, Lymphocyte-activation protein 3; TIGIT, T-cell Immunoreceptor With Ig And ITIM Domains; VISTA, V-domain Ig suppressor of T-cell activation; DNMT, DNA Methyltransferase; HDAC, Histone deacetylase; HPV, human papillomavirus; PI3K, Phosphoinositide 3-kinase; CSF1R, Colony-stimulating factor 1 receptor; NFKB, nuclear factor kappa B; JAK, Janus kinase; TGF, Transforming growth factor.

**Genes/pathways very important in the pathogenesis of antibody deficiencies.

*Genes/pathways important in the pathogenesis of antibody deficiencies.

## Author Contributions

HA, YW, LH, and QP-H equally contributed to the design and writing of the manuscript. All authors contributed to the article and approved the submitted version.

## Funding

This work was supported by the Swedish Research Council, the Swedish Cancer Society (Cancerfonden), the Swedish Childhood Cancer Fund, Radiumhemmets, the Center for Innovative Medicine, Jonas Söderquist scholarship, and Åke Wibergs stiftelse.

## Conflict of Interest

The authors declare that the research was conducted in the absence of any commercial or financial relationships that could be construed as a potential conflict of interest.

## Publisher’s Note

All claims expressed in this article are solely those of the authors and do not necessarily represent those of their affiliated organizations, or those of the publisher, the editors and the reviewers. Any product that may be evaluated in this article, or claim that may be made by its manufacturer, is not guaranteed or endorsed by the publisher.
